# Epigrass: a tool to study disease spread in complex networks

**DOI:** 10.1186/1751-0473-3-3

**Published:** 2008-02-26

**Authors:** Flávio C Coelho, Oswaldo G Cruz, Cláudia T Codeço

**Affiliations:** 1 Programa de Computação Científica, Fundação Oswaldo Cruz, Rio de Janeiro, Brasil

## Abstract

**Background:**

The construction of complex spatial simulation models such as those used in network epidemiology, is a daunting task due to the large amount of data involved in their parameterization. Such data, which frequently resides on large geo-referenced databases, has to be processed and assigned to the various components of the model. All this just to construct the model, then it still has to be simulated and analyzed under different epidemiological scenarios. This workflow can only be achieved efficiently by computational tools that can automate most, if not all, these time-consuming tasks. In this paper, we present a simulation software, Epigrass, aimed to help designing and simulating network-epidemic models with any kind of node behavior.

**Results:**

A Network epidemiological model representing the spread of a directly transmitted disease through a bus-transportation network connecting mid-size cities in Brazil. Results show that the topological context of the starting point of the epidemic is of great importance from both control and preventive perspectives.

**Conclusion:**

Epigrass is shown to facilitate greatly the construction, simulation and analysis of complex network models. The output of model results in standard GIS file formats facilitate the post-processing and analysis of results by means of sophisticated GIS software.

## Background

Epidemic models describe the spread of infectious diseases in populations. More and more, these models are being used for predicting, understanding and developing control strategies. To be used in specific contexts, modeling approaches have shifted from "strategic models" (where a caricature of real processes is modeled in order to emphasize first principles) to "tactical models" (detailed representations of real situations). Tactical models are useful for cost-benefit and scenario analyses. Good examples are the foot-and-mouth epidemic models for UK, triggered by the need of a response to the 2001 epidemic [1,2] and the simulation of pandemic flu in different scenarios helping authorities to choose among alternative intervention strategies [3,4].

In realistic epidemic models, a key issue to consider is the representation of the contact process through which a disease is spread, and network models have arisen as good candidates [5]. This has led to the development of "network epidemic models". Network is a flexible concept that can be used to describe, for example, a collection of individuals linked by sexual partnerships [6], a collection of families linked by sharing workplaces/schools [7], a collection of cities linked by air routes [8]. Any of these scales may be relevant to the study and control of disease spread [9].

Networks are made of nodes and their connections. One may classify network epidemic models according to node behavior. One example would be a classification based on the states assumed by the nodes: networks with discrete-state nodes have nodes characterized by a discrete variable representing its epidemiological status (for example, susceptible, infected, recovered). The state of a node changes in response to the state of neighbor nodes, as defined by the network topology and a set of transmission rules. Networks with continuous-state nodes, on the other hand, have node' state described by a quantitative variable (number of susceptibles, density of infected individuals, for example), modelled as a function of the history of the node and its neighbors. The importance of the concept of neighborhood on any kind of network epidemic model stems from its large overlap with the concept of transmission. In network epidemic models, transmission either defines or is defined/constrained by the neighborhood structure. In the latter case, a neighborhood structure is given a priori which will influence transmissibility between nodes.

The construction of complex simulation models such as those used in network epidemic models, is a daunting task due to the large amount of data involved in their parameterization. Such data frequently resides on large geo-referenced databases. This data has to be processed and assigned to the various components of the model. All this just to construct the model, then it still has to be simulated, analyzed under different epidemiological scenarios. This workflow can only be achieved efficiently by computational tools that can automate most if not all of these time-consuming tasks.

In this paper, we present a simulation software, Epigrass, aimed to help designing and simulating network-epidemic models with any kind of node behavior. Without such a tool, implementing network epidemic models is not a simple task, requiring a reasonably good knowledge of programming. We expect that this software will stimulate the use and development of networks models for epidemiological purposes. The paper is organized as following: first we describe the software and how it is organized with a brief overview of its functionality. Then we demonstrate its use with an example. The example simulates the spread of a directly transmitted infectious disease in Brazil through its transportation network. The velocity of spread of new diseases in a network of susceptible populations depends on their spatial distribution, size, susceptibility and patterns of contact. In a spatial scale, climate and environment may also impact the dynamics of geographical spread as it introduces temporal and spatial heterogeneity. Understanding and predicting the direction and velocity of an invasion wave is key for emergency preparedness.

### Epigrass

Epigrass is a platform for network epidemiological simulation and analysis. It enables researchers to perform comprehensive spatio-temporal simulations incorporating epidemiological data and models for disease transmission and control in order to create complex scenario analyses. Epigrass is designed towards facilitating the construction and simulation of large scale metapopulational models. Each component population of such a metapopulational model is assumed to be connected through a contact network which determines migration flows between populations. This connectivity model can be easily adapted to represent any type of adjacency structure.

**Figure 1:**
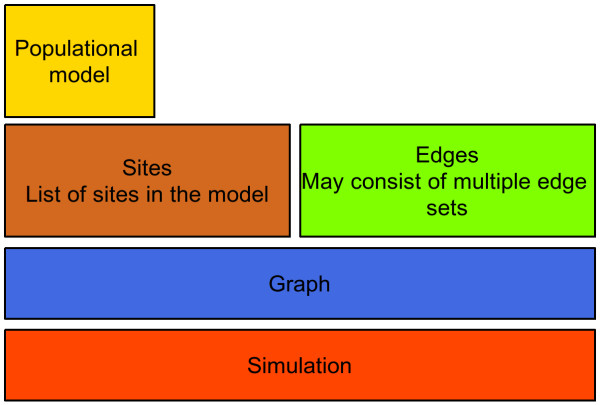
**Architecture of an Epigrass simulation model.** A simulation object contains the whole model and all other objects representing the graph, sites and edges. Site object contaim model objects, which can be one of the built-in epidemiological models or a custom model written by the user.

Epigrass is entirely written in the Python language, which contributes greatly to the flexibility of the whole system due to the dynamical nature of the language. The geo-referenced networks over which epidemiological processes take place can be very straightforwardly represented in a object-oriented framework. Consequently, the nodes and edges of the geographical networks are objects with their own attributes and methods (figure 1).

Once the archetypal node and edge objects are defined with appropriate attributes and methods, then a code representation of the real system can be constructed, where nodes (representing people or localities) and contact routes are instances of node and edge objects, respectively. The whole network is also an object with its own set of attributes and methods. In fact, Epigrass also allows for multiple edge sets in order to represent multiple contact networks in a single model.

These features leads to a compact and hierarchical computational model consisting of a network object containing a variable number of node and edge objects. It also does not pose limitations to encapsulation, potentially allowing for networks within networks, if desirable. This representation can also be easily distributed over a computational grid or cluster, if the dependency structure of the whole model does not prevent it (this feature is currently being implemented and will be available on a future release of Epigrass). For the end-user, this hierarchical, object-oriented representation is not an obstacle since it reflects the natural structure of the real system. Even after the model is converted into a code object, all of its component objects remain accessible to one another, facilitating the exchange of information between all levels of the model, a feature the user can easily include in his/her custom models.

Nodes and edges are dynamical objects in the sense that they can be modified at runtime altering their behavior in response to user defined events. In Epigrass it is very easy to simulate any dynamical system embedded in a network. However, it was designed with epidemiological models in mind. This goal led to the inclusion of a collection of built-in epidemic models which can be readily used for the intra-node dynamics (SIR model family). Epigrass users are not limited to basing their simulations on the built-in models. User-defined models can be developed in just a few lines of Python code. All simulations in Epigrass are done in discrete-time. However, custom models may implement finer dynamics within each time step, by implementing ODE models at the nodes, for instance.

**Figure 2:**
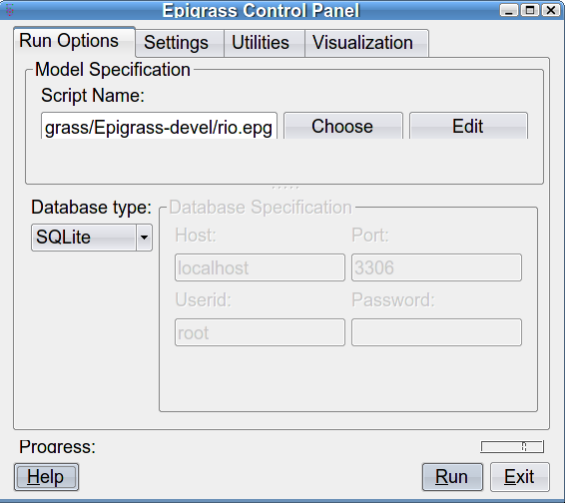
Epigrass graphical user interface.

The Epigrass system is driven by a graphical user interface(GUI), which handles several input files required for model definition and manages the simulation and output generation (figure 2).

**Figure 3:**
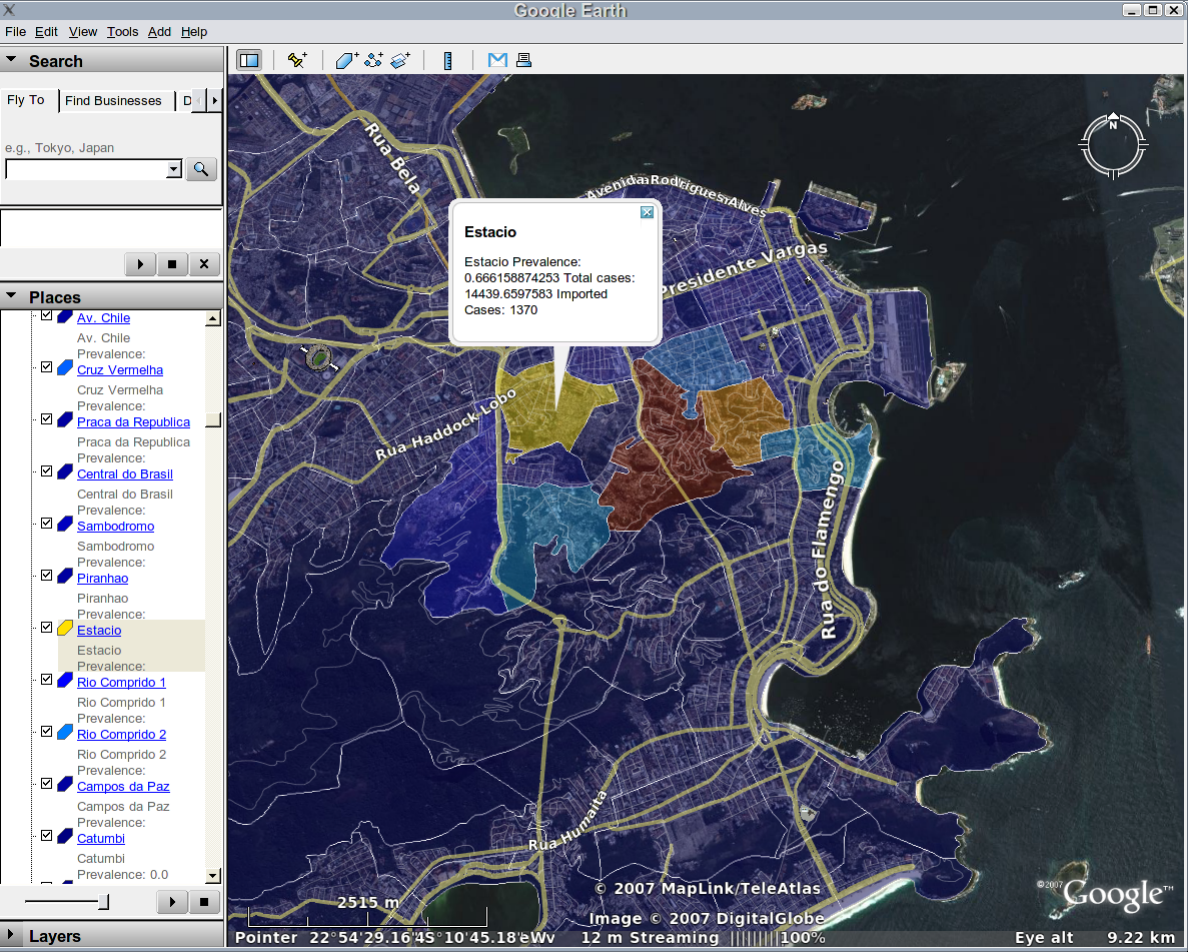
**Workflow for a typical Epigrass simulation.** This diagram shows all inputs and outputs typical of an Epigrass simulation session.

At the core of the system lies the simulator. It parses the model specification files, contained in a text file (.epg file), and builds the network from site and edge description files (comma separated values text files, CSV). The simulator then builds a code representation of the entire model, simulates it, and stores the results in the database or in a couple of CSV files. This output will contain the full time series of the variables in the model. Additionally, a map layer (in shapefile and KML format) is also generated with summary statitics for the model (figure 3).

**Figure 4:**
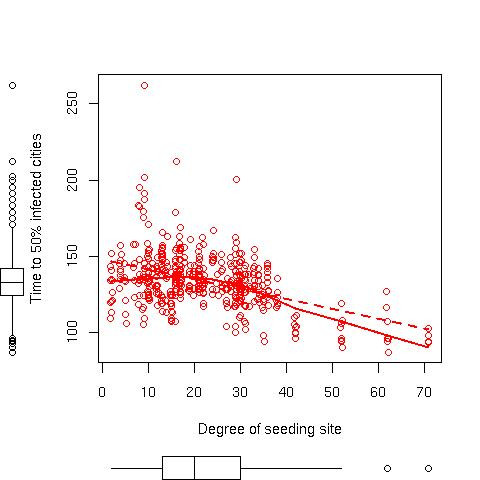
**Epigrass animation output. **Sites are color coded (from red to blue) according to infection times. Bright red is the seed site (on the NE).

**Figure 5:**
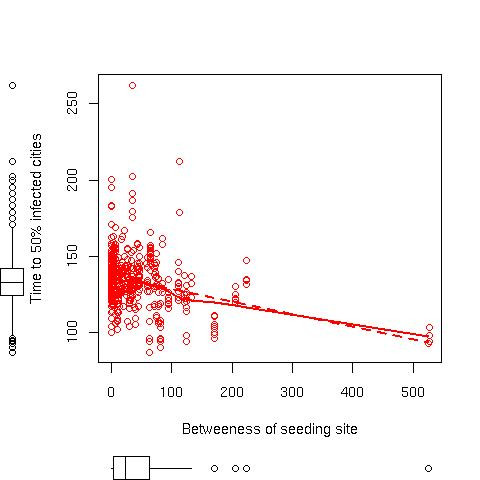
Epigrass output visualized on Google-Earth.

The results of an Epigrass simulation can be visualized in different ways. A map with an animation of the resulting timeseries is available directly through the GUI (figure 4). Other types of static visualizations can be generated through GIS software from the shapefiles generated. The KML file can also be viewed in Google Earth™ or Google Maps™ (figure 5).

Epigrass also includes a report generator module which is controlled through a parameter in the ".epg" file.

Epigrass is capable of generating PDF reports with summary statistics from the simulation. This module requires a LATEX installation to work. Reports are most useful for general verification of expected model behavior and network structure. However, the LATEX source files generated by the module may serve as templates that the user can edit to generate a more complete document.

Building a model in Epigrass is very simple, especially if the user chooses to use one of the built-in models. Epigrass includes 20 different epidemic models ready to be used (See manual for built-in models description).

To run a network epidemic model in Epigrass, the user is required to provide three separate text files (Optionally, also a shapefile with the map layer):

1. Node-specification file: This file can be edited on a spreadsheet and saved as a csv file. Each row is a node and the columns are variables describing the node.

2. Edge-specification file: This is also a spreadsheet-like file with an edge per row. Columns contain flow variables.

3. Model-specification file: Also referred to as the ".epg" file. This file specifies the epidemiological model to be run at the nodes, its parameters, flow model for the edges, and general parameters of the simulation.

The ".epg" file is normally modified from templates included with Epigrass. Nodes and edges files on the other hand, have to be built from scratch for every new network. Details of how to construct these files, as well as examples, can be found in the documentation accompanying the software, which is available at at the project's website [10]

## Methods

### Example model

In the example application, the spread of a respiratory disease through a network of cities connected by bus transportation routes is analyzed.

The epidemiological scenario is one of the invasion of a new influenza-like virus. One may want to simulate the spread of this disease through the country by the transportation network to evaluate alternative intervention strategies (e.g. different vaccination strategies). In this problem, a network can be defined as a set of nodes and links where nodes represent cities and links represents transportation routes. Some examples of this kind of model are available in the literature [8,11].

One possible objective of this model is to understand how the spread of such a disease may be affected by the point-of-entry of the disease in the network. To that end, we may look at variables such as the speed of the epidemic, number of cases after a fixed amount of time, the distribution of cases in time and the path taken by the spread.

The example network was built from 76 of largest cities of Brazil (>= 100 k habs). The bus routes between those cities formed the connections between the nodes of the networks. The number of edges in the network, derived from the bus routes, is 850. These bus routes are registered with the National Agency of Terrestrial Transportation (ANTT) which provided the data used to parameterize the edges of the network.

### The model

The epidemiological model used consisted of a metapopulation system with a discrete-time SEIR model (Eq. 1). For each city, *S*_*t *_is the number of susceptibles in the city at time *t*, *E*_*t *_is the number of infected but not yet infectious individuals, *I*_*t *_is the number of infectious individuals resident in the locality, *N *is the population residing in the locality (assumed constant throughout the simulation), and *n*_*t *_is the number of individuals visiting the locality, Θ_*t *_is the number of visitors who are infectious. The parameters used were taken from Lipsitch et al. (2003) [12] to represent a disease like SARS with an estimated basic reproduction number (*R*_0_) of 2.2 to 3.6 (Table 1).

**Table 1:** Parameters used in the models and their meaning. Parameter n and *θ *have their values derived stochatically during the simularion, therefore their values are not given here.

**Symbol**	**Meaning**.	**Value**
*β*	contact rate (*t*^-1^)	[1.4, 2.27]^1^
*θ*	number of infectious visitors	
*α*	mixing parameter	1
*n*	number of visitors	
*N*	population (*S *+ *E *+ *I *+ *R*)	city population
*r*	fraction of *I *recovering from infection (*day*^-1^)	0.2
*e*	fraction of *E *becoming infectious (*day*^-1^)	0.2

$$\begin{array}{c} S_{t+1}=S_{t}-\beta S_{t}\frac{{\left(I_{t}+\Theta \right)}^{\alpha }}{N_{t}+n_{t}}\\ E_{t+1}=(1-e)E_{t}+\beta S_{t}\frac{{\left(I_{t}+\Theta_{t}\right)}^{\alpha }}{N_{t}+n_{t}}\\ I_{t+1}=eE_{t}+(1-r)I_{t}\\ R_{t+1}=N_{t}-(S_{t+1}+I_{t+1}+E_{t+1}) \end{array}$$

To simulate the spread of infection between cities, we used the concept of a "forest fire" model [13]. An infected individual, traveling to another city, acts as a spark that may trigger an epidemic in the new locality. This approach is based on the assumption that individuals commute between localities and contribute temporarily to the number of infected in the new locality, but not to its demography. Implications of this approach are discussed in Grenfell et al (2001) [13].

The number of individuals arriving in a city (*n*_*t*_) is based on annual total number of passengers arriving trough all bus routes leading to that city as provided by the ANTT (Brazilian National Agency for Terrestrial Transportation). The annual number of passengers is used to derive an average daily number of passengers simply by dividing it by 365.

Stochasticity is introduced in the model at two points: the number of new cases is draw from a Poisson distribution with intensity $\frac{{\left(I_{t}+\Theta_{t}\right)}^{\alpha }}{N_{t}+n_{t}}$ and the number of infected individuals visiting *i *is modelled as binomial process:

$$\begin{array}{lll} \Theta_{t} & = & \sum_{k}\theta_{k,t}\text{ for all k neighbors}\\ \theta_{k,t} & \sim & Binomial\left(n,\frac{I_{k,t-\delta }}{N_{k}}\right) \end{array}$$

where *n *is the total number of passengers arriving from a given neighboring city; *I*_*k, t *_and *N*_*k *_are the current number of infectious individuals and the total population size of city *k*, respectively. *δ *is the delay associated with the duration of each bus trip. The delay *δ *was calculated as the number of days (rounded down) that a bus, traveling at an average speed of 60 *km/h*, would take to complete a given trip. The lengths in kilometers of all bus routes were also obtained from the ANTT.

**Figure 6:**
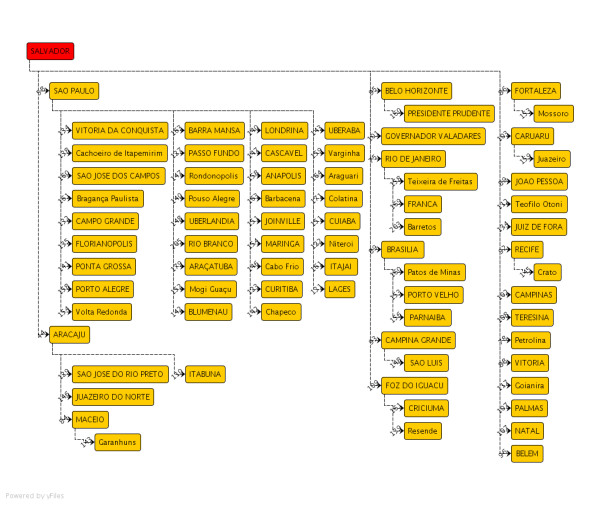
Cost in vaccines applied vs. benefit in cases avoided, for a simulated epidemic starting at the highest degree city (São Paulo).

**Figure 7:**
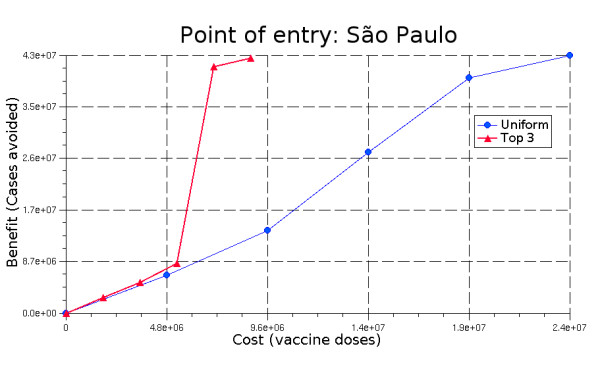
Cost in vaccines applied vs. benefit in cases avoided, for a simulated epidemic starting at a relatively low degree city(Salvador).

Vaccination campaigns in specific (or all) cities can be easily attained in Epigrass, with individual coverages for each campaign on each city. We use this feature to explore Vaccination scenarios in this model (figures 6 and 7).

The files with this model's definition(the sites, edges and ".epg" files) are available as part of the Additional files 1, 2 and 3 for this article.

### Analysis

To determine the importance of the point of entry in the outcome of the epidemic, the model was run 500 times, randomizing the point of entry of the virus. The seeding site was chosen with a probability proportional to the *log*_10 _of their population size. These replicates were run using Epigrass' built-in support for repeated runs with the option of randomizing seeding site. For every simulation, statistics about each site such as the time it got infected and time series of incidence were saved.

The time required for the epidemic to infect 50% of the cities was chosen as a global index to network susceptibility to invasion. To compare the relative exposure of cities to disease invasion, we also calculated the inverse of time elapsed from the beginning of the epidemic until the city registered its first indigenous case as a local measure of exposure.

Except for population size, all other epidemiological parameters were the same for all cities, that is, disease transmissibility and recovery rate. Some positional features of each node were also derived: *Centrality*, which is is a measure derived from the average distance of a given site to every other site in the network; *Betweeness*, which is the number of times a node figures in the the shortest path between any other pair of nodes; and *Degree*, which is the number of edges connected to a node.

**Figure 8:**
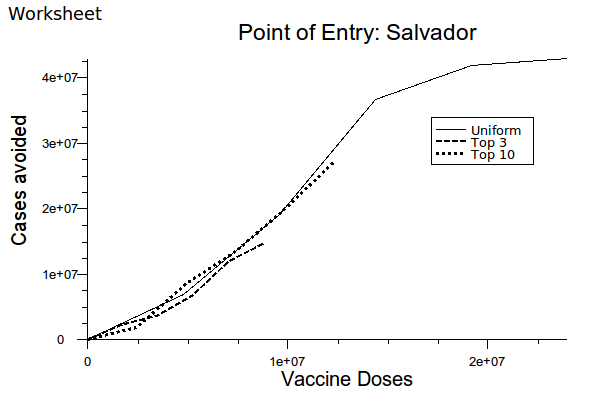
**Spread of the epidemic starting at the city of Salvador, a city with relatively small degree (that is, small number of neighbors).** The number next to the boxes indicated the day when each city developed its first indigenous case.

In order to analyze the path of the epidemic spread, we also recorded which cities provided the infectious cases which were responsible for the infection of each other city. If more than one source of infection exists, Epigrass selects the city which contributed with the largest number of infectious individuals at that time-step, as the most likely infector. At the end of the simulation Epigrass generates a file with the dispersion tree in graphML format, which can be read by a variety of graph plotting programs to generate the graphic seen on figure 8.

## Results and discussion

### Scalability of epigrass

The computational cost of running a single time step in an epigrass model, is mainly determined by the cost of calculating the epidemiological models on each site(node). Therefore, time required to run models based on larger networks should scale linearly with the size of the network (order of the graph), for simulations of the same duration. The model presented here, took 2.6 seconds for a 100 days run, on a 2.1 GHz cpu. A somewhat larger model with 343 sites and 8735 edges took 28 seconds for a 100 days simulation. Very large networks may be limited by the ammount of RAM available. The authors are working on adapting Epigrass to distribute processing among multiple cpus(in SMP systems), or multiple computers in a cluster system. The memory demands can also be addressed by keeping the simulation objects on an object-oriented database during the simulation. Steps in this direction are also being taken by the development team.

### Epidemiological analyses

**Figure 9:**
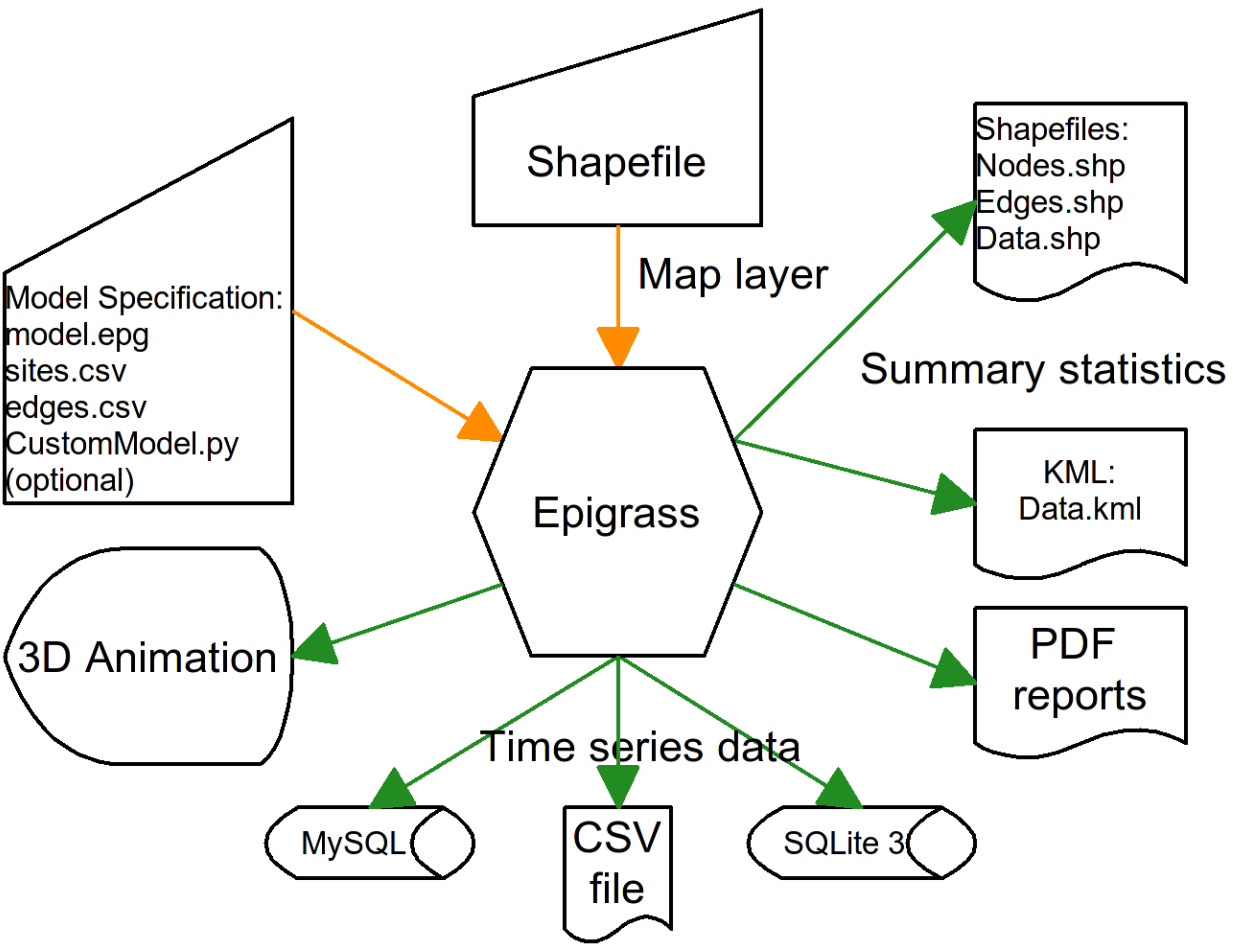
Effect of degree(a) and betweeness(b) of entry node to the speed of the epidemic.

**Figure 10:**
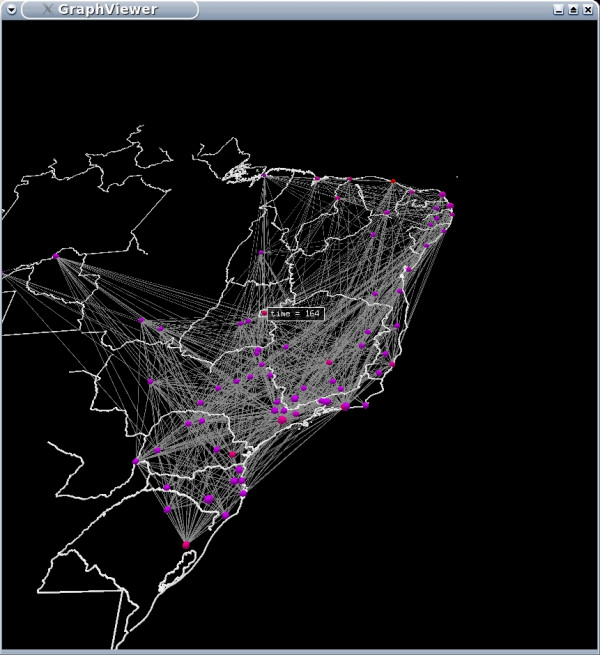
Effect of betweeness of entry node on the speed of the epidemic.

The model presented here served maily the purpose of illustrating the capabilities of Epigrass for simulating and analyzing reasonably complex epidemic scenarios. It should not be taken as a careful and complete analysis of a real epidemic. Despite that, some features of the simulated epidemic are worth discussing. For example: The spread speed of the epidemic, measured as the time taken to infect 50% of the cities, was found to be influenced by the centrality and degree of the entry node (figures 9 and 10). The dispersion tree corresponding to the epidemic, is greatly influenced by the degree of the point of entry of the disease in the network. Figure 8 shows the tree for the dispersion from the city of Salvador.

Vaccination strategies must take into consideration network topology. Figures 6 and 7 show cost benefit plots for three vaccination strategies investigated: *Uniform vaccination, top-3 degree sites only *and *top-10 degree sites only*. Vaccination of higher order sites offer cost/benefit advantages only in scenarios where the disease enter the network through one of these sites.

## Conclusion

Epigrass facilitates greatly the simulation and analysis of complex network models. The output of model results in standard GIS file formats facilitates the post-processing and analysis of results by means of sophisticated GIS software. The non-trivial task of specifying the network over which the model will be run, is left to the user. But epigrass allows this structure to be provided as a simple list of sites and edges on text files, which can easily be contructed by the user using a spreadsheet, with no need for special software tools.

Besides invasion, network epidemiological models can also be used to understand patterns of geographical spread of endemic diseases [14-17]. Many infectious diseases can only be maintained in a endemic state in cities with population size above a threshold, or under appropriate environmental conditions(climate, availability of a reservoir, vectors, etc). The variables and the magnitudes associated with endemicity threshold depends on the natural history of the disease [18]. Theses magnitudes may vary from place to place as it depends on the contact structure of the individuals. Predicting which cities are sources for the endemicity and understanding the path of recurrent traveling waves may help us to design optimal surveillance and control strategies.

## Authors' contributions

FCC contributed with the software development, model definition and analysis as well as general manuscript conception and writing. CTC contributed with model definition and implementation, as well as with writing the manuscript. OGC, contributed with data analysis and writing the manuscript. All authors have read and approved the final version of the manuscript.

## Supplementary Material

Additional file 1 Click here for file

Additional file 2 Click here for file

Additional file 3 Click here for file
